# Overexpression of *NtWRKY50* Increases Resistance to *Ralstonia solanacearum* and Alters Salicylic Acid and Jasmonic Acid Production in Tobacco

**DOI:** 10.3389/fpls.2017.01710

**Published:** 2017-10-11

**Authors:** Qiuping Liu, Ying Liu, Yuanman Tang, Juanni Chen, Wei Ding

**Affiliations:** College of Plant Protection, Southwest University, Chongqing, China

**Keywords:** NtWRKY50, salicylic acid, jasmonic acid, *Ralstonia solanacearum*, disease resistance

## Abstract

WRKY transcription factors (TFs) modulate plant responses to biotic and abiotic stresses. Here, we characterized a WRKY IIc TF, NtWRKY50, isolated from tobacco (*Nicotiana tabacum*) plants. The results showed that NtWRKY50 is a nuclear-localized protein and that its gene transcript is induced in tobacco when inoculated with the pathogenic bacterium *Ralstonia solanacearum*. Overexpression of *NtWRKY50* enhanced bacterial resistance, which correlated with enhanced SA and JA/ET signaling genes. However, silencing of the *NtWRKY50* gene had no obvious effects on plant disease resistance, implying functional redundancy of NtWRKY50 with other TFs. In addition, it was found that *NtWRKY50* can be induced by various biotic or abiotic stresses, such as Potato virus Y, *Rhizoctonia solani, Phytophthora parasitica*, hydrogen peroxide, heat, cold, and wounding as well as the hormones salicylic acid (SA), jasmonic acid (JA), and ethylene (ET). Importantly, additional analysis suggests that *NtWRKY50* overexpression markedly promotes SA levels but prevents pathogen-induced JA production. These data indicate that *NtWRKY50* overexpression leads to altered SA and JA content, increased expression of defense-related genes and enhanced plant resistance to *R. solanacearum.* These probably due to increased activity of endogenous *NtWRKY50* gene or could be gain-of-function phenotypes by altering the profile of genes affected by *NtWRKY50*.

## Introduction

Plants exhibit a set of innate immune system responses during interaction with various pathogens. Disease resistance mechanisms consist of two-tiered defense responses: the first layer is involves plant transmembrane pattern recognition receptors (PRRs) detecting pathogen-associated molecular patterns (PAMPs) to induce PAMP-triggered immunity (PTI), and the second involves plant resistance (R) proteins targeting pathogen avirulence effector proteins to induce effector-triggered immunity (ETI) (Chisholm et al., 2006; Jones and Dangl, 2006). In particular, PTI and ETI are involved into plant systemic acquired resistance (SAR), a long-distance defense reaction (Durrant and Dong, 2004). In plants, local and systemic defense responses are mediated by the signaling pathways of the interconnected phytohormones salicylic acid (SA), jasmonic acid (JA), and ethylene (ET) (Bostock, 2005). It is also known that SA plays an important signaling molecule role implicated in plant disease resistance to biotrophs (Kunkel and Brooks, 2002), and JA and ET pathways mediate plant defense against necrotrophs at the early stage of infection (Glazebrook, 2005).

WRKY transcription factors (TFs), which compose a major protein family in plants, contain ∼60-amino acid-long DNA binding domains (DBDs) and zinc-finger motifs. WRKYs are divided into groups I, II, and III based on their DBD and zinc-finger motifs, and group II is further divided into IIa, IIb, IIc, IId and IIe, resulting from the primary amino acid sequence (Eulgem et al., 2000; Xie et al., 2005; Zhang and Wang, 2005). WRKYs directly interact with W-box elements (with the core motif TTGACC/T in the promoter) of downstream genes to regulate various signaling pathways (Eulgem et al., 2000).

WRKY proteins play a vital role in mediating the expression of a large number of genes by binding to W-box elements in the promoter region during pathogen infection, which correlates with the modulation of SA and JA signaling pathways in most cases. It has been shown that the up-regulation of *Arabidopsis* WRKY70 promotes SA-dependent defense signaling but represses JA defense signaling. *WRKY70* overexpression shows enhanced resistance to the bacterial necrotroph *Erwinia carotovora*, the fungal biotroph *Erysiphe cichoracearum* and the hemibiotrophic *Pseudomonas syringae* but enhanced susceptibility to the fungal necrotroph *Alternaria brassicicola* (Li et al., 2004, 2006). The *Atwrky70* mutant displays reduced plant resistance to *E. cichoracearum* but increased resistance to *A. brassicicola* (Li et al., 2006). Another report has demonstrated that the *Arabidopsis Atwrky18Atwrky40* and *Atwrky18Atwrky60* double mutants and the *Atwrky18Atwrky40Atwrky60* triple mutants are more resistant to the hemibiotrophic bacterial pathogen *P. syringae* but more susceptible to the necrotrophic fungal pathogen *Botrytis cinerea*. Expression of SA-responsive *Pathogenesis-Related1* (*PR1*) and JA-regulated *Plant-Defensin1.2* (*PDF1.2*) is consistently oppositely affected in these mutants in response to the two pathogens. Interestingly, overexpression of *AtWRKY18* together with *AtWRKY40* or *AtWRKY60* promotes plants susceptibility to both *P. syringae* and *B. cinerea* (Xu et al., 2006). This also suggests that a complex and overlapping regulatory network exists between WRKY TFs. Overexpression of *AtWRKY38* and *AtWRKY62* has been shown to negatively affect plant resistance toward *P. syringae* and to suppress SA-regulated *PR1* gene expression (Kim et al., 2008). However, another study reported that *AtWRKY62* overexpression inhibits JA-responsive gene transcripts (Mao et al., 2007). Overall, these findings indicate different functions of WRKY TFs in SA- and JA/ET-mediated plant defenses.

WRKY proteins have been shown to act as positive and negative regulators of plant resistance against *Ralstonia solanacearum*, a soilborne bacterium that infects numerous crops, including potato, tomato, tobacco, peanut, eggplant, and banana. *Arabidopsis* RRS1 (AtWRKY52), which contains a typical toll-interleukin1-receptor nucleotide binding site, leucine-rich repeat R protein motifs and a WRKY domain, confers resistance to *R. solanacearum.* Deslandes et al. (2002) found that the AtWRKY52/RRSI-R protein in *Arabidopsis* mediates resistance to *R. solanacearum*. In addition to having a typical signal for nuclear localization and a WRKY domain, AtWRKY52 also contain an NBS-LRR domain of R genes. Therefore, *AtWRKY52* functions both as a typical plant disease resistance gene involved in resistance to the invasion of the pathogenic bacterium *R. solanacearum* and as a typical WRKY gene involved in signal transduction in *Arabidopsis* resistance (Lahaye, 2002; Deslandes and Marco, 2003). Recently, it was shown that *Capsicum annuum* CaWRKY6, CaWRKY40, and CaWRKY27 promote plant disease resistance to *R. solanacearum* (Dang et al., 2014; Cai et al., 2015). In contrast, CaWRKY58 inhibits plant resistance toward this pathogen (Wang et al., 2013).

In this study, a deduced WRKY gene, termed *NtWRKY50*, was isolated from tobacco plants. To understand the biological function of NtWRKY50, we characterized NtWRKY50 via sequence comparison, subcellular localization, pathogenicity analysis, and expression of defense-related genes in *NtWRKY50* transgenic plants.

## Results

### Expression of a *WRKY*-Like Gene during *R. solanacearum* Infection

We found that a *WRKY*-like gene was significantly activated in tobacco (*Nicotiana tabacum*) under *R. solanacearum* infection (unpublished data) based on preliminary transcriptomic data (unpublished data). We thus designed primers according to the predicted CDS sequence for qRT-PCR analysis. The ubiquitin gene is among the most stable genes in tobacco (Rotenberg et al., 2006) and is commonly used as internal reference gene in QRT-PCR. To roughly confirm the stability of ubiquitin gene *UBI3* under various stress conditions, the expressions of *WRKY* gene were analyzed using three reference genes *EF1α* (Rotenberg et al., 2006), *ACT9* (Rotenberg et al., 2006) and *UBI3*, or one reference gene UBI3. The results indicate the transcript levels of WRKY gene were quite similar between two analyses suggesting *UBI* expressions were quite stable under different conditions (Supplementary Figure S1). Hence, *UBI3* was used as qRT-PCR reference gene.

**FIGURE 1:**
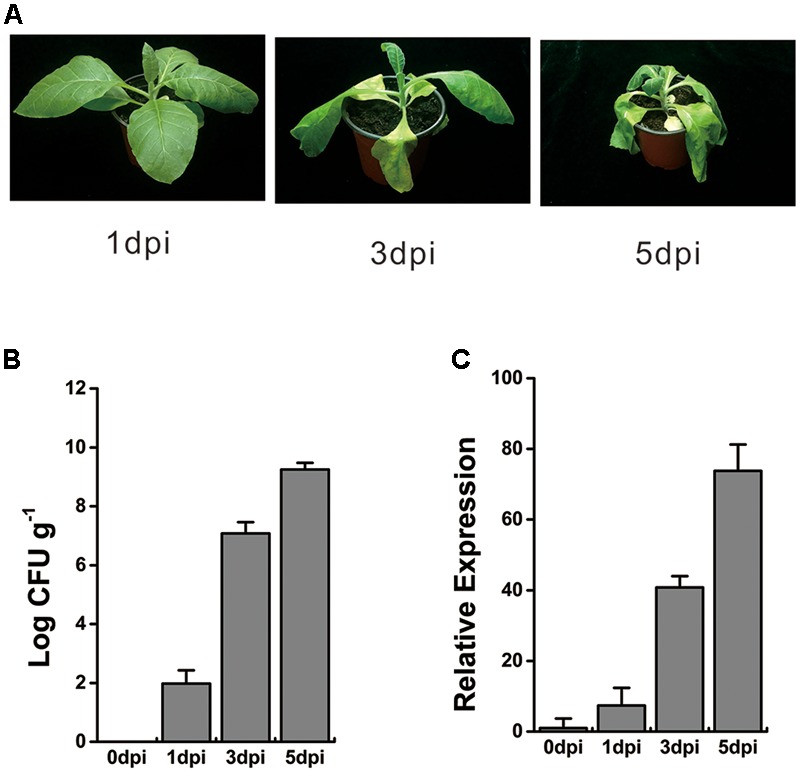
Expression profile of *WRKY*-like gene in response to *Ralstonia solanacearum* infection. **(A)** The symptoms of WT tobacco plants inoculated with *R. solanacearum*. Roots of 8-week-old WT plants were wounded and inoculated with 10 ml (1 × 10^8^ CFU ml^-1^) of *R. solanacearum* CQPS-1 cells, and images were taken at 1, 3, and 5 days post inoculation (dpi). **(B)** Growth of *R. solanacearum* in the roots of WT at 0, 1, 3, and 5 dpi. Plants were root wounded and inoculated as in **(A)**. **(C)** Relative expression level of *WRKY*-like gene in WT plants inoculated with *R. solanacearum*. The inoculation was performed as in **(A)**. Roots and base stem were pooled to extract the total RNA at 0, 1, 3, and 5 dpi respectively. The *UBI3* was used as a reference gene, and the transcript level at 0 dpi was set to a value of ‘1.’

To verify the transcriptional profiles of this *WRKY*-like gene under *R. solanacearum* infection, 8-week-old tobacco plants were inoculated with 10 ml of bacterial cells at a concentration of 10^8^ colony-forming units (CFU) ml^-1^ via wounding of the roots. At 1 day post inoculation (dpi), no wilt symptoms were observed, and very few bacterial titers were detected in tobacco roots. However, the bacterial titers significantly increased, and the wilt symptoms became more serious at 3 and 5 dpi (**Figures 1A,B**). The whole roots and stem base of inoculated plant were harvested at 0, 1, 3, and 5 dpi, respectively. Total RNA was extracted, and cDNA was further generated by reverse transcription. The qRT-PCR analysis showed that the expression of *WRKY*-like gene was induced at 1 dpi and increased over time, until 5 dpi (**Figure 1C**). These results suggest a consistent and increasing activation of *WRKY*-like gene expression during *R. solanacearum* infection.

### Phylogenic Analysis of NtWRKY50

**FIGURE 2:**
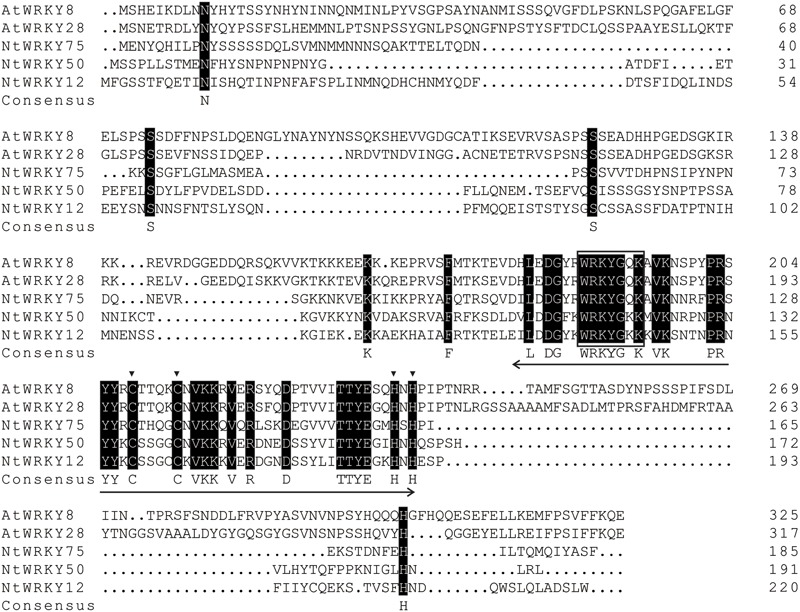
Sequence analysis of the deduced NtWRKY50 protein. Identical amino acids are shaded black. The approximately 60-amino acid-long WRKY domain is marked with a two-headed arrow, and the C and H residues in the zinc-finger motif are marked with triangles. The highly conserved amino acid sequence WRKYGKK in the WRKY domain is boxed. The following proteins were used for analysis: AtWRKY8 (NP_199447.1), AtWRKY28 (NP_193551.1), NtWRKY75 (XP_016497198.1), NtWRKY12 (NP_001312362.1).

A pair of primers were designed, and polymerase chain reaction was performed to isolate this WRKY-like gene. The results showed that a sequence of 576 base pairs encoding 191 amino acids was amplified. Nucleotide BLAST demonstrated that the deduced WRKY gene shows 100% similarity to *NtWRKY50.* Then, this protein was termed NtWRKY50. The encoded NtWRKY50 protein contains the WRKY domain WRKYGKK, which contains a substitution of G to K in the typical WRKYGQK sequence, and a zinc-finger motif (C-X_4-5_-C-X_22-23_-H-X_1_-H) (**Figure 2**).

**FIGURE 3:**
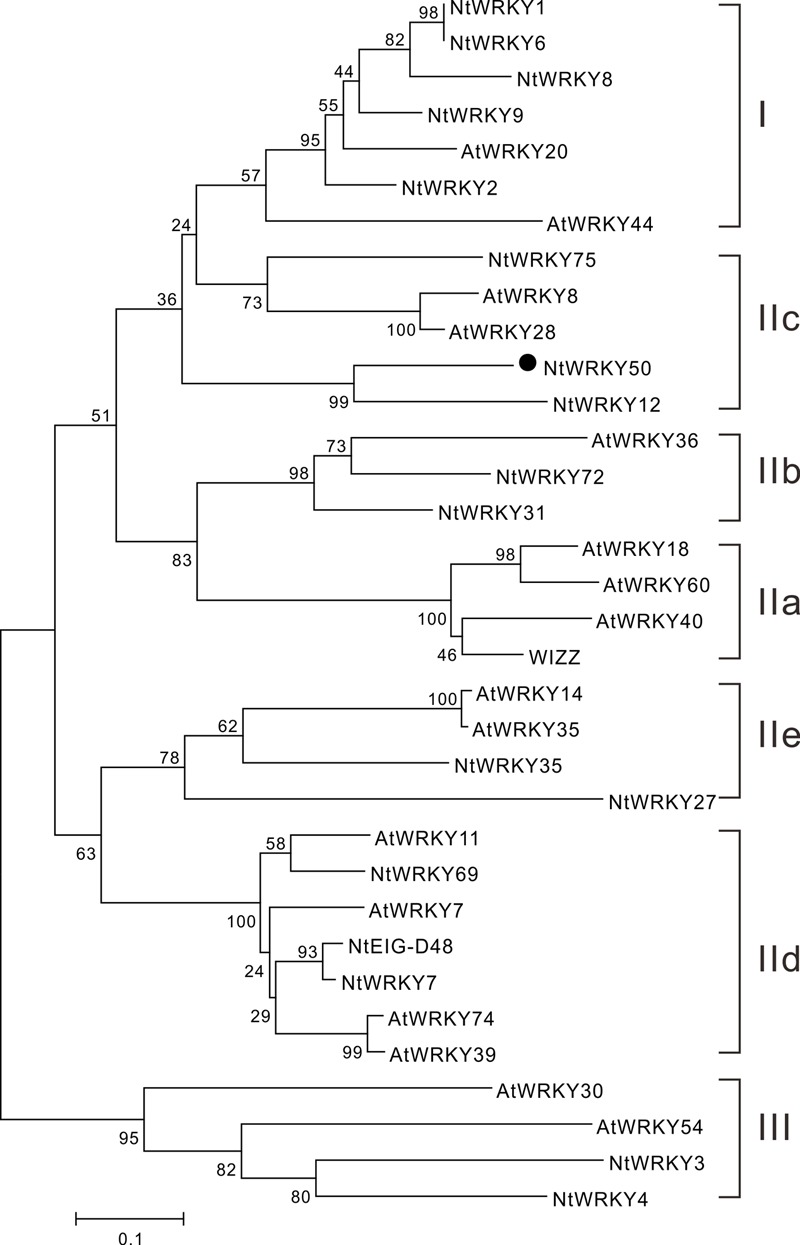
Phylogenetic analysis of the NtWRKY50 protein among WRKY proteins from *Arabidopsis* and tobacco. The neighbor-joining phylogenetic tree was created with Clustal W using MEGA5.1. NtWRKY50 is highlighted with black circle. The numbers above or below branches indicate bootstrap values (>50%) from 1000 replicates. The species origins of the WRKYs are indicated by abbreviation before the gene names: At, *Arabidopsis thaliana*; Nt, *Nicotiana tabacum*. The following proteins were used for analysis: AtWRKY18 (NP_001329847.1), AtWRKY7 (NP_194155.1), AtWRKY11 (NP_849559.10), AtWRKY74 (NP_198217.1), AtWRKY39 (NP_566236.1), AtWRKY60 (AASequence:1009113411 from TAIR), AtWRKY36 (NP_564976.1), AtWRKY8 (NP_199447.1), AtWRKY28 (NP_193551.1), AtWRKY14 (NP_564359.1), AtWRKY35 (NP_181029.1), AtWRKY44 (NP_181263.2), AtWRKY20 (NP_849450.1), AtWRKY30 (NP_568439.1), AtWRKY54 (NP_181607.1), NtWRKY3 (NP_001312512.1), NtWRKY1 (XP_016482650.1), NtEIG-D48 (NP_001312390.1), NtWRKY-8 (NP_001311829.1), NtWRKY12 (NP_001312362.1), NtWRKY-6 (NP_001311970.1), NtWRKY-9 (XP_016456273.1), WIZZ (NP_001312579.1), WRKY2 (NP_001312319), WRKY4 (XP_016459189.1), WRKY69 (XP_016500385.1), NtWRKY27 (XP_016506104.1), NtWRKY75 (XP_016497198.1), NtWRKY7 (XP_016498573.1), NtWRKY31 (XP_016458925.1), NtWRKY35 (XP_016470169.1), NtWRKY72 (XP_016502535.1) were used to construct the phylogenetic tree. Notably, the sequence for AtWRKY60 was obtained from *Arabidopsis* database TAIR and others were from National Center for Biotechnology Information (NCBI).

We next performed a phylogenetic analysis for NtWRKY50 using MEGA 5.1 and the neighbor-joining method. The results showed that WRKYs were generally divided into three categories: WRKY I, WRKY II, and WRKY III. The WRKY II category was further divided into five subgroups, including IIa, IIb, IIc, IId, and IIe. NtWRKY50, along with *Arabidopsis thaliana* AtWRKY8 and AtWRKY28, was classified into the IIc subgroup (**Figure 3**). This subfamily was purported to contain a large amount of variation in the WRKYGQK heptapeptide sequence, and this variation was consistent with our results (**Figure 2**) (Xiang et al., 2016).

### Localization of NtWRKY50

**FIGURE 4:**
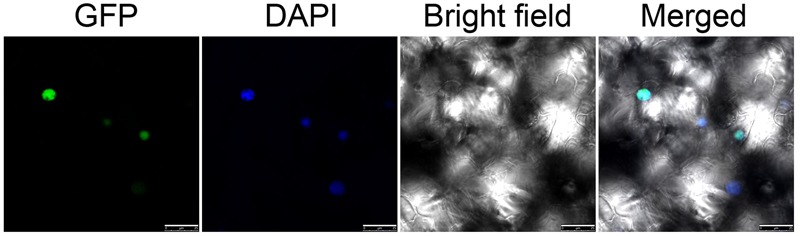
Subcellular localization of NtWRKY50. Transient expression of 35S::NtWRKY50::GFP constructs in tobacco leaf cells. Green fluorescence corresponding to the expressed proteins was observed with a fluorescence microscope 24 h after infiltration. The nuclei of the tobacco cells were visualized by DAPI staining.

To investigate the localization of NtWRKY50, the open reading frame sequence of *NtWRKY50* was fused in frame to the C-terminus of a green fluorescent protein (GFP) gene controlled by the cauliflower mosaic virus CaMV35S promoter in a pEGAD vector. Using *Agrobacterium*-induced transient expression in the leaves of tobacco, we observed that GFP-fused NtWRKY50 localized in the nucleus of leaf epidermal cells (**Figure 4**).

### Overexpression of *NtWRKY50* Promotes Plant Disease Resistance

**FIGURE 5:**
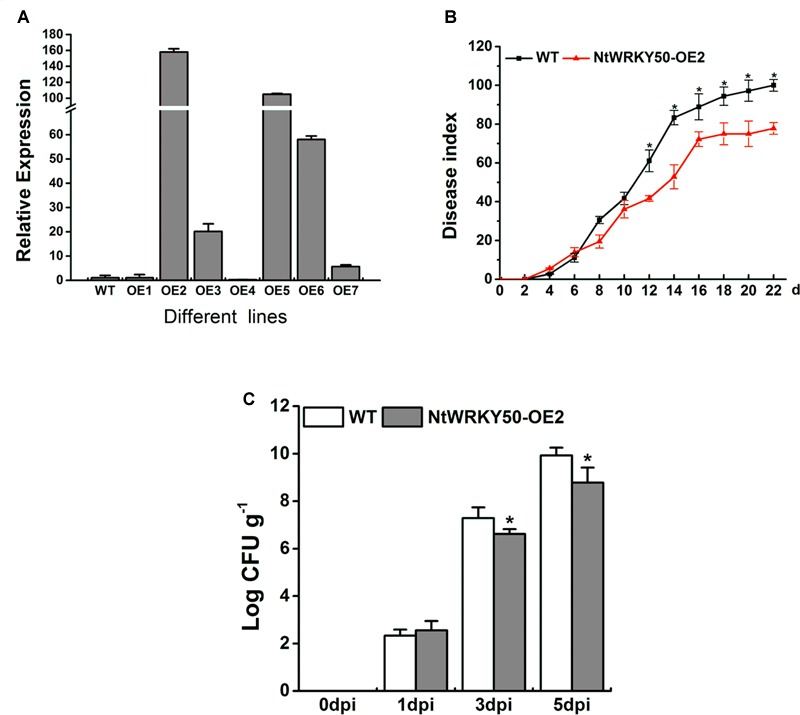
Overexpression of *NtWRKY50* enhanced tobacco resistance to *R. solanacearum* infection. **(A)** Relative expression level of *NtWRKY50* in different *NtWRKY50*-overexpressing transgenic lines. **(B)** Disease index of NtWRKY50-OE2 and WT plants infected by *R. solanacearum*. Ten plants were used for each line and were inoculated with 10 ml of *R. solanacearum* cells by root irrigation. **(C)** Growth of *R. solanacearum* in the roots of NtWRKY50-OE2 and WT at 0, 1, 3, and 5 dpi. Plants were root wounded and inoculated as in B. Error bars indicate the SE of three independent experiments. Asterisks above bars indicate statistically significant (*t-*test, *P* < 0.05) differences between NtWRKY50-OE2 and WT using SPASS version 13.0 software.

**FIGURE 6:**
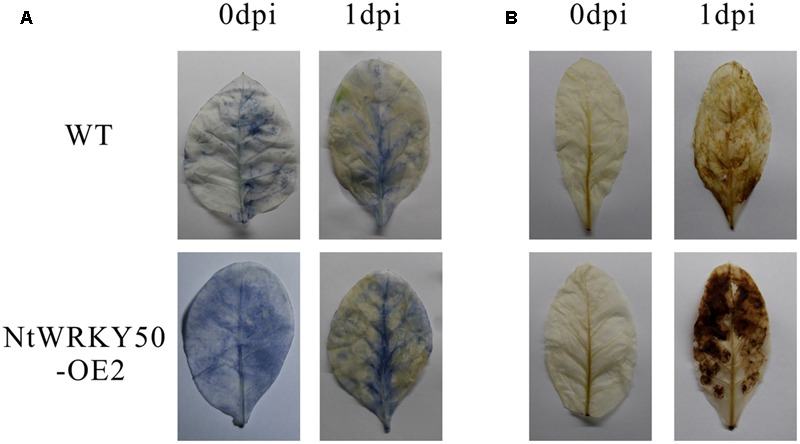
Histochemical staining. DAB **(A)** and NBT **(B)** staining of WT and the *NtWRKY50* overexpression line NtWRKY50-OE2 before inoculation (0 dpi) and 1 day post inoculation (1 dpi).

To investigate the role of *NtWRKY50* in plant disease defense, we generated *NtWRKY50*-overexpression transgenic tobacco plants, in which the *NtWRKY50* expression is controlled via the cauliflower mosaic virus CaMV35S promoter. Among seven transgenic tobacco lines, the OE2 line (NtWRKY50-OE2) showed a higher expression of *NtWRKY50* and was used for the pathogenicity analysis (**Figure 5A**). When 8-week-old tobacco plants were inoculated with 10 ml of *R. solanacearum* cells at a concentration of 10^8^ CFU ml^-1^ by root irrigation. NtWRKY50-OE2 plants were more resistant than WT and presented lower disease incidence (**Figure 5B**). To assess the bacterial growth, roots and base stems were collected from plants inoculated via the root-wounding method. The results indicate the bacterial growth was influenced and was significantly lower in NtWRKY50-OE2 than in WT (**Figure 5C**). In addition, DAB (3, 3′-diaminobenzidine) and NBT (nitro blue tetrazolium) staining showed stronger ROS in the NtWRKY50-OE2 plants (**Figure 6**). Thus, these results suggest that NtWRKY50 positively regulates tobacco resistance to *R. solanacearum*.

### Silencing of *NtWRKY50* Shows No Effects on Plant Disease Resistance

To further determine the role of *NtWRKY50* in regulating plant disease resistance, we used the antisense silencing technique to knock down the expression of *NtWRKY50* in tobacco. The transcript levels of *NtWRKY50* in the silenced lines and WT tobacco plants were detected using qRT-PCR. The results showed that the S2 line had the lowest transcription levels of *NtWRKY50* (Supplementary Figure S2A). Then, S2 plants were inoculated with *R. solanacearum* cells as were the *NtWRKY50-*overexpression plants. The result showed that there was no difference in bacterial titers and disease symptoms between *NtWRKY50*-silenced lines and WT (Supplementary Figures S2B,C). These results indicate that silencing *NtWRKY50* does not alter tobacco disease resistance.

### *NtWRKY50* Regulates Hormone-Related Defense Signaling

**FIGURE 7:**
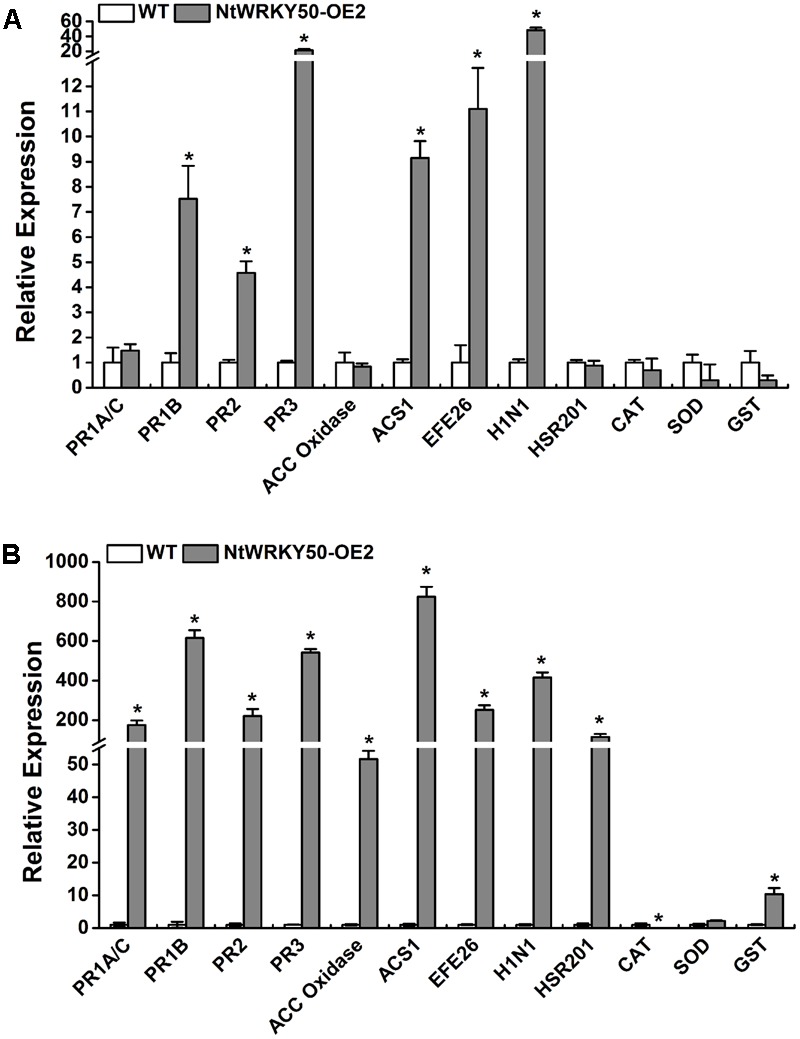
qRT-PCR analysis of defense-related genes before and after inoculation with *R. solanacearum*. qRT-PCR was performed before **(A)** and 1 day post inoculation **(B)**. Plant roots were wounded and inoculated with 10 ml of *R. solanacearum* cells by root irrigation. Roots and base stem were pooled for total RNA extraction for NtWRKY50-OE2 and WT plants, respectively. The *UBI3* gene was used as a reference gene. Asterisks represent significant differences (*t-*test, *P* < 0.05) using SPASS version 13.0 software.

Salicylic acid, JA, and ET are important defense signaling molecules. We next detected the effects of NtWRKY50 overexpression on the expression of SA-, JA-, and ET-responsive genes. Total RNA was extracted from both 8-week-old NtWRKY50-OE2 and WT plants before and after *R. solanacearum* inoculation, and cDNA was generated. SA- and JA/ET-responsive PR genes, such as *PR1A/C, PR1B, PR2, PR3*; ET biosynthesis and signaling marker genes, including *ACC oxidase, ACS1*, and *EFE26*; HR-related genes *HSR201* and *H1N1*; and genes encoding ROS-scavenging enzymes, including catalase (CAT), superoxide dismutase (SOD), and glutathione *S*-transferase (GST), were analyzed using qRT-PCR. Before pathogen infection, 3–6-fold up-regulation was observed for the gene expression of *PR1B, PR2, ACS1, EFE26* in NtWRKY50-OE2 line compared with WT line, while *PR3* and *H1N1* increased 20–30 fold (**Figure 7A**). Other genes did not show significant changes. However, the transcript levels of the majority of genes in NtWRKY50-OE2 were 50–800-fold more abundant than in WT after pathogen infection (**Figure 7B**). ROS-scavenging enzymes exhibited different regulatory patterns: CAT decreased, while GST was up-regulated 10 fold in the NtWRKY50-OE2 line compared to WT line. These findings indicate *NtWRKY50* may induce hormone-mediated defense pathways, especially after pathogen infection.

### Plant Hormones and Biotic and Abiotic Stresses Induce *NtWRKY50* Transcripts

**FIGURE 8:**
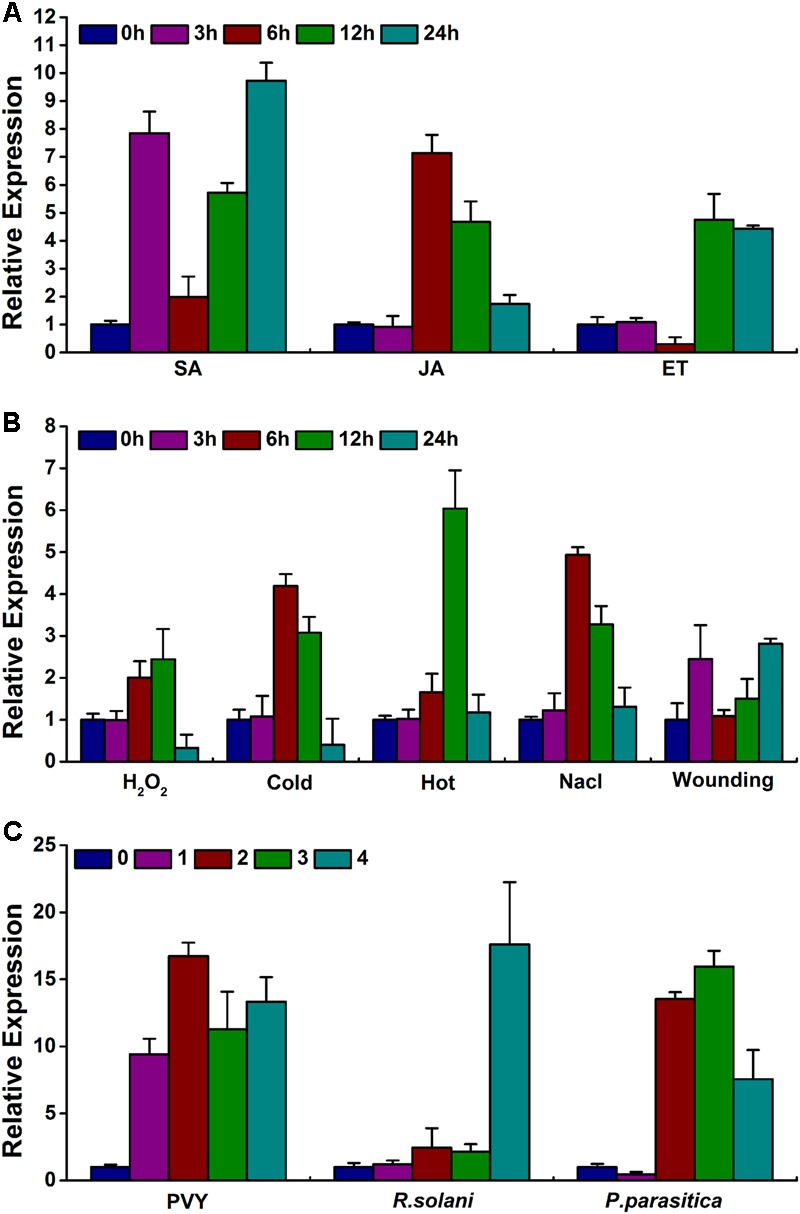
Expression pattern of *NtWRKY50* under various hormone and stress conditions. **(A)** *NtWRKY50* expression under hormone treatments. The following hormones were used: SA, JA, and ET. **(B)** *NtWRKY50* expression under abiotic stresses treatments. The following stresses were used: hydrogen peroxidase (H_2_O_2_), NaCl, heat (38°C) and cold (14°C). **(C)** *NtWRKY50* expression under biotic stress treatments. The following pathogens were used: Potato virus Y (PVY), *Rhizoctonia solani* (*R. solani*) and *Phytophthora parasitica* (*P. parasitica*). Eight-week-old WT plants were used for treatments.

To investigate whether *NtWRKY50* is altered by various plant hormones and abiotic stresses, 8-week-old WT plants were treated with the plant hormones SA, JA and ET and with abiotic stresses, such as hydrogen peroxide (H_2_O_2_), heat, cold, NaCl, and wounding. Materials were harvested at indicated time points. The results showed that *NtWRKY50* expression increased markedly to the highest value (10 and 7 fold) at 24 and 6 h after spraying with SA and JA, respectively (**Figure 8A**). When treated with ET, *NtWRKY50* increased by approximately 5 fold at 12 h, which was its highest peak. Other abiotic stress conditions also led to the up-regulation of *NtWRKY50* (**Figure 8B**). Cold, heat, and NaCl treatments resulted in 4–6-fold more expression of *NtWRKY50* at 12, 6, and 6 h, respectively. Wounding and H_2_O_2_ treatments showed the most inconspicuous induction; the highest value was the double of that of the control.

WRKY genes probably respond to multiple plant pathogens. Therefore, *NtWRKY50* expression in response to different types of pathogens was tested. *Phytophthora parasitica* (*P. parasitica*), *Rhizoctonia solani* (*R. solani*) and Potato virus Y (PVY), which belong to oomycetes, fungi and viruses, respectively, were applied to leaves of tobacco. *NtWRKY50* transcripts were also activated under various pathogen infections, although the expression patterns were distinct (**Figure 8C**), indicating that *NtWRKY50* gene expression responds to environmental changes.

### *NtWRKY50* Promotes SA Levels but Prevents Pathogen-Induced JA Accumulation

**FIGURE 9:**
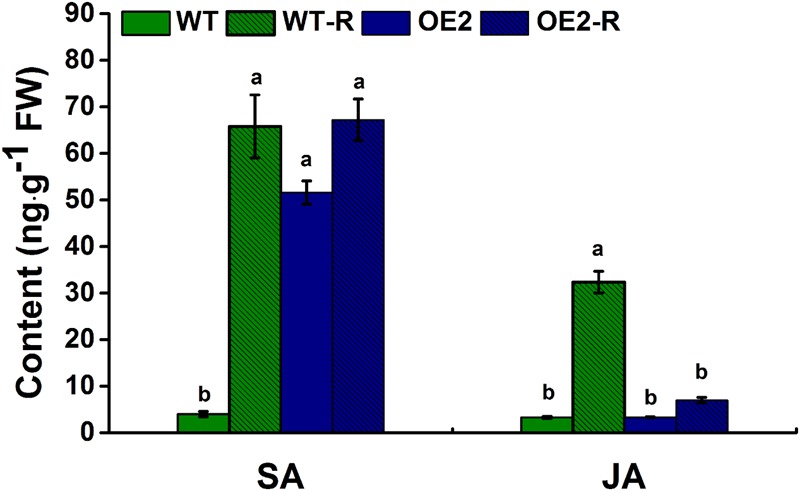
Endogenous levels of free SA and JA. Free SA and JA were quantitatively analyzed using high-performance liquid chromatography–mass spectrometry according to the procedure described in the materials and methods. ‘WT-R’ and ‘OE2-R’ represent WT and NtWRKY50-OE2 infected with *R. solanacearum* respectively. Different letters above the columns indicate significant differences (*P* < 0.05) according to least-significant-difference (LSD) test performed using SPASS version 13.0 software.

Regarding the strong morphological phenotypes observed in overexpression plants, one possibility to explain the mode of action of NtWRKY50 is that this TF might be involved in endogenous hormone biosynthesis. To test this possibility, we determined whether the endogenous levels of hormones implicated in plant defense were affected in *NtWRKY50* transgenic plants. Therefore, the levels of SA and JA, both of which are important in defense responses, were determined in NtWRKY50-OE2 and WT plants before and after *R. solanacearum* inoculation. As shown in **Figure 9**, a small amount of SA was detected in WT, whereas NtWRKY50-OE2 possessed a relatively high amount of SA before pathogen inoculation (**Figure 9**). However, after infection by *R. solanacearum*, SA levels increased dramatically in WT but stayed unchanged in NtWRKY50-OE2. The amount of JA was similar between WT and NtWRKY50-OE2 before pathogen infection and JA increased markedly to a high level in WT but barely changed in NtWRKY50-OE2 (**Figure 9**). These findings suggest *NtWRKY50* influences SA and JA amounts in the presence and absence of pathogen invasion.

## Discussion

WRKY TFs play important roles in modulating plant immune responses. Increasing evidence has demonstrated that some WRKYs affect disease resistance when overexpressed or silenced in plants (Eulgem and Somssich, 2007). Here, we found that the WRKY IIc protein NtWRKY50 localizes to the nucleus.

In our preliminary study, we found that the soilborne bacterium *R. solanacearum* significantly induces the gene expression of *NtWRKY50* in tobacco plants (unpublished data). Thus, we speculated the role of *NtWRKY50* in plant disease resistance and performed a pathogenicity analysis. The results showed that overexpression of *NtWRKY50* prevents the growth of *R. solanacearum* and the development of disease symptoms in tobacco plants (**Figure 5**). Many WRKY proteins have been shown to affect plant immunity as negative regulators, such as AtWRKY4 (Lai et al., 2008), AtWRKY11 (Journot-Catalino and Kroj, 2006), AtWRKY17 (Journot-Catalino and Kroj, 2006), AtWRKY27 (Mukhtar et al., 2008), CaWRKY1 and CaWRKY58 (Wang et al., 2013), and GhWRKY40 (Wang et al., 2014) or as positive regulators, such as CaWRKY40 (Dang et al., 2013), CaWRKY27 (Dang et al., 2014), and CaWRKY6 (Cai et al., 2015). These data indicate a positive role for *NtWRKY50* in plant defense. However, loss of NtWRKY50 causes no change in resistance to tobacco plants against *R. solanacearum*, suggesting that NtWRKY50 may be functionally redundant with other TFs (**Figure 6**); functional redundancy is an inherent feature of WRKY genes (Eulgem and Somssich, 2007; Pandey and Somssich, 2009).

A burst of reactive oxygen species (ROS), consisting of mostly H_2_O_2_ and $O_{2}^{•–}$, occurs in plants during pathogen infection (Bhattacharjee, 2005). The H_2_O_2_ from the oxidative burst is associated with the hypersensitive response (HR) of programmed cell death (Levine et al., 1994; Van Breusegem and Dat, 2006); an increase in the HR is an indicator of enhanced accumulation of ROS (Torres et al., 2006). Our results showed that ROS levels and gene transcripts of HR-associated H1N1 were enhanced in NtWRKY50-OE2 lines in response to *R. solanacearum* infection (**Figures 7, 8**), resulting in improved plant resistance. ROS have dual roles in plant–pathogen interactions: on one hand, they stimulate host resistance reactions (Low and Merida, 1996) or kill the pathogen directly (Peng and Kuc, 1992); on the other hand, they lead to plant harm (Sutherland, 1991; Elstner and Osswald, 1994; Baker and Orlandi, 1995). Plant cells have a protection system that allows the generation and clearing of ROS in a dynamic equilibrium: SOD, CAT, and GST play a role in eliminating excess cell ROS and oxidizing substances. Our data demonstrate GST was significantly elevated in NtWRKY50-OE2 compared with WT (**Figure 7**). However, it is still unclear why CAT levels decreased in NtWRKY50-OE2. Although SA is capable of binding to CAT and GST, which results in the inhibition of their activities (Tian et al., 2012), SA has also been shown to enhance the expression of GST (Shirasu et al., 1997). In addition, increased levels of SA have been shown to potentiate ROS accumulation (Siegrist et al., 1994; Fauth et al., 1996), which would activate the synthesis of more SA through self-amplification loops, involving H_2_O_2_ accumulation, SA synthesis, and cell death (Shirasu et al., 1997). These phenomena were consistent with our results, in that ROS levels and SA content were both elevated in the NtWRKY50-OE2 line (**Figures 6, 9**).

Salicylic acid, JA, and ET are important molecules in the signal transduction pathways of plant disease resistance (Robert-Seilaniantz et al., 2007). WRKY TFs have been indicated to play important roles in the regulation of hormone-dependent defense signaling (Li et al., 2004; Kim et al., 2008). In this study, SA- and JA/ET-responsive PR genes as well as ET synthesis and signaling genes were up-regulated in NtWRKY50-OE2 line up to 30 fold before *R. solanacearum* infection and up to 50–800 fold after *R. solanacearum* infection compared to levels in WT (**Figure 7**). Interestingly, ET-insensitive tobacco plants are less resistant to necrotrophic pathogens (Geraats et al., 2003). Previous studies have suggested that SA mainly mediates the resistance of plants to biotrophic pathogens, whereas JA/ET mediates the resistance to necrotrophic pathogens and insects and that the two pathways generally act antagonistically. However, after tomato is infected with *P. syringae*, tomato resistance against pathogens and insects is enhanced, and the feeding of insects promotes tomato resistance against pathogens. This finding suggests that the SA pathway and the JA/ET pathway can also act synergistically (Stout et al., 1999). These results seem to support the notion that *NtWRKY50* improves SA and JA/ET defense pathways together. Consistent with above results, *NtWRKY50* could be significantly induced by SA, JA, and ET (**Figure 8**).

*NtWRKY50* also responded to multiple external stimuli, indicating a role of *NtWRKY50* in plant basal defense (**Figure 8**). The plant hormones SA and JA are essential for plants to cope with numerous environmental stresses, such as temperature fluctuations, water and nutrient imbalances, and pathogens (Santner and Estelle, 2009; Wang et al., 2009). It is likely that *NtWRKY50* influences plant responses to environmental factors by modifying hormone-mediated signaling pathways.

Hormone analysis indicated that *NtWRKY50* overexpression specially promotes the production of SA (10 fold) but prevents JA accumulation after pathogen infection (**Figure 9**). There is crosstalk between SA and JA, and high concentrations of SA and JA have antagonistic effects on each other (Mur et al., 2006). However, these antagonistic effects do not occur in systemic tissue or even in adjacent tissue if an ETI occurs (Spoel et al., 2007). In this study, the high basal level of SA in NtWRKY50-OE2 may inhibit JA accumulation after pathogen infection in contrast to WT, in which SA and JA both presented low concentrations before inoculation and increased together during pathogen infection.

Salicylic acid is a critical factor in potentiating a variety of defense responses, including the expression of defense genes and the deposition of cell wall phenolics in pathogen-treated plants (Siegrist et al., 1994). Several varieties of plants and various mutants that contain increased SA levels are highly resistant to pathogen attack (Yalpani and Raskin, 1993; Yang et al., 1997). Moreover, SA is suggested to be necessary and sufficient to induce SAR, which enhances plant resistance to a broad range of pathogens (Gaffney et al., 1993; Vernooij et al., 1994; Wildermuth et al., 2001). The key difference between resistant and susceptible plants is that a resistant plant is capable of rapidly deploying a wide variety of defense responses that prevent pathogen colonization; in contrast, a susceptible plant exhibits much weaker and slower responses that fail to restrict pathogen growth and/or spread (Yang et al., 1997). Therefore, high levels of basal SA in NtWRKY50-OE2 may prime the plants for pathogen attack, resulting in stronger defense responses, such as higher ROS content (**Figure 6**) and increased transcripts of hormone-related defense genes relative to those in WT (**Figure 7**), thus enhancing plant resistance (**Figure 5**).

It is unknown how *NtWRKY50* affects SA levels. One explanation is that *NtWRKY50* may affect components in the SA synthesis pathway (Zheng and Dong, 2013). WRKY *ics* elements and W-boxes are present in the promoter region of the SA synthetase gene ICS1 (Maleck et al., 2000; Wildermuth et al., 2001; Yu et al., 2001). *Arabidopsis WRKY28, WRKY46*, and *WRKY54* have been reported to modify ICS1 accumulation and SA levels (Wang et al., 2006; van Verk et al., 2011). A second explanation is that *NtWRKY50* affects SA signaling events. Similar to ICS1, W-boxes are also overrepresented in the promoter of NPR1, the master regulator of the SA signaling pathway (Cao et al., 1997). Two *Arabidopsis* WRKYs, WRKY38 and WRKY 62, are associated with the degradation of NPR1 (Spoel et al., 2009). Since *NPR1* seems to feedback regulate SA biosynthesis (Wang et al., 2006; Zheng and Dong, 2013), *NtWRKY50* might affect SA amounts by regulating SA signaling components. *NtWRKY50* could be induced by SA treatment, suggesting that it acts downstream of SA. Therefore, the second explanation is much more plausible.

Two possibilities should be addressed regarding the mode of NtWRKY50 function. First, WRKY genes exhibit extensive autoregulation and cross-regulation and are functionally connected, forming a complex transcriptional web (Eulgem and Somssich, 2007). Moreover, WRKY proteins function through a diverse array of protein interaction partners, such as MAP kinases, calmodulin, histone deacetylases, and resistance proteins (Rushton et al., 2010). A single WRKY TF might regulate several different processes. Hence, NtWRKY50 probably acts indirectly through other TF genes. Second, due to the particular mode of action, overexpressing a TF usually results in two types of gain of function: hypermorphy (high form) and neomorphy (new form). The introduced gene may display the same function as the endogenous gene but at a higher activity level (high form) or might display a new function (new form) because of improper expression (Zhang, 2003). Therefore, the phenotype of NtWRKYOE2, i.e., increased ROS and SA levels, unchanged JA levels after pathogen inoculation and enhanced expression of defense genes, could also be the result of a new function due to improper expression.

### Conclusion

Plants recognize pathogens through a series of signal transduction processes, leading to the final induction of plant defense genes. In these processes, WRKY TFs play an important role in activating or inhibiting the expression of defense genes through interaction with other related proteins. At the same time, the WRKY TF itself is also regulated by other factors. Changes in expression levels of specific WRKY TFs greatly alter stress resistance. In this study, we identified and characterized a tobacco WRKY member belonging to group IIc. These findings suggest that constitutive overexpression of *NtWRKY50* promotes tobacco resistance to the bacterial pathogen *R. solanacearum*. Loss-of-function data indicate a redundancy feature of NtWRKY50 with other TFs. Notably, *NtWRKY50* overexpression led to a marked increase in SA production but inhibited pathogen-induced JA accumulation; on the other hand, transcripts of *NtWRKY50* were markedly induced by SA and JA, indicating an important role in the SA and JA regulatory networks. Promotion of *NtWRKY50* expression by various abiotic stresses and pathogens might be a subsequent result from the effects of this TF on hormone production and suggests this protein is involved in basal responses to environmental stimuli.

## Materials and Methods

### Pathogens, Plants, and Growth Conditions

*Ralstonia solanacearum* (*R. solanasearum*) isolate CQPS-1 (race 1, biovar 3, phylotype I, sequevar 17) was cultivated at 30°C. Plants of tobacco (*Nicotiana tabacum*) cultivar Yunyan87 [wild type (WT)] were grown in a growth chamber at 28°C and 70% relative humidity under a 14-h photoperiod.

### Subcellular Localization

The full-length coding region of *NtWRKY50* was inserted into the XmaI and BamHI restriction sites of pEGAD, after which the resultant plasmid or empty control plasmid were transformed into *Agrobacterium tumefaciens* strain EHA105.

### Generation of Transgenic Plants

The coding sequence of *NtWRKY50* was predicted from transcriptome sequencing results. The primers used in this study are listed in Supplementary Table S1. The coding sequence was inserted into the BglII and BstEII restriction sites of the plant expression vector pVCT2024 or Eco31I site of pBWA(V)HS to generate overexpression lines and RNAi lines, respectively, under the control of the CaMV35S promoter. Tobacco transformation was accomplished according to the procedures of Horsch et al. (1985). Transgenic plants were selected using kanamycin (100 mg L^-1^) and confirmed using PCR. Positive plants were propagated asexually in Murashige Skoog (MS) medium and then transferred to soil; plants at the 8-leaf stage were ready for bacterial inoculation. WT Yunyan87 tobacco plants were also cultivated in MS medium, propagated and transplanted at the same time as the transgenic tobacco plants and served as controls for *R. solanacearum* inoculation and other analyses.

### *Ralstonia solanacearum* Inoculation and Growth Assessment

*Ralstonia solanacearum* was grown in B liquid medium (Vasse et al., 2000) for 16 h at 30°C during shaking. Bacteria were then collected by centrifugation and resuspended in distilled water.

For the disease incidence analysis, 10 transgenic and WT plants were root inoculated with 10 ml (1 × 10^8^ CFU ml^-1^) of *R. solanacearum* cells. The experiment was repeated three times. Disease grades were as follows: 0, no wilting; 1, 1–25% wilted; 2, 26–50% wilted; 3, 51–75% wilted; and 4, 76–100% wilted or dead. The experiment was repeated three times.

For the bacterial growth assays, transgenic and WT plants were wounded on the roots and inoculated with 10 ml (1 × 10^8^ CFU ml^-1^) of *R. solanacearum* cells. Roots (three plants were pooled into one sample) were sampled at each time point of bacterial inoculation, weighed and then rinsed with distilled water five times before being ground using 10 ml of distilled water. Dilutions of bacteria were spotted on B agar medium, and colonies were counted after 2 days, which allowed the bacterial density (CFU g^-1^) in the roots to be calculated. The experiment was repeated three times. The Student’s *t*-test was then used to determine significant differences between the transgenic plants and WT plants.

### RNA Extraction and Quantitative RT-PCR

The total RNA of tobacco plants was extracted from roots and base stem using TRIzol^®^ Reagent (Invitrogen, Carlsbad, CA, United States). The RNA concentration and integrity were then analyzed using an Agilent Bioanalyzer 2100 system (Agilent Technologies, Santa Clara, CA, United States).

qRT-PCR was performed using a C1000 Touch^TM^ Thermal Cycler (BIO-RAD, United States) and a CFX96^TM^ real-time system, with SsoFAST^TM^ Eva Green Supermix. Transcripts of genes were standardized using *UBI3* (Schmidt and Delaney, 2010). Primers used in this assay were designed using Primer 5.0 software (Premier Biosoft International) and are listed in Supplementary Table S1. PCR amplification was performed according to the following conditions: 95°C for 3 min; 40 cycles of 95°C for 10 s, 55°C for 15 s, 72°C for 15 s; and a melt cycle from 65 to 95°C.

### Histochemical Staining

Before and 1 day after *R. solanacearum* inoculation, the leaves of WT and NtWRKY50-OE2 plants were stained with 3, 3′-diaminobenzidine (DAB, 1 mg ml^-1^) or nitro blue tetrazolium (NBT, 0.1 mg ml^-1^) for 24 and 6 h, respectively, at 25°C in the dark. The DAB-stained leaves were cleared by boiling in [lactic:glycerol:absolute ethanol (1:1:3, V:V:V)] and then destained overnight in absolute ethanol (Korasick et al., 2010). The NBT-stained leaves were destained overnight in absolute ethanol directly. After destaining, the leaves were soaked and preserved in fresh ethanol at room temperature and imaged.

### Biotic and Abiotic Stress Treatments

Eight-week-old WT plants were sprayed with 2 mM SA, 0.1 mM JA, 7 mM ET, 10 mM hydrogen peroxidase (H_2_O_2_) or 200 mM NaCl. Plants were incubated at 38 or 15°C for heat and cold treatments, respectively. Wounding consisted of eight parallel cuts caused by a blade.

For biotic stress treatments, 8-week-old WT plants were mechanically inoculated with potato virus Y (PVY) or were wounded using a scalpel, after which the leaves were inoculated with *Phytophthora parasitica* or *Rhizoctonia solani*. Samples of PVY-infected plants were collected at 0, 6, 10, 11, and 12 dpi, whereas the plants infected with the other two pathogens were harvested at 0, 1, 2, 3, and 4 dpi. Three plants were pooled into one sample for qRT-PCR analysis, and the experiment was repeated three times.

### Analysis of Plant Hormones

Free SA and JA were quantitatively analyzed using high-performance liquid chromatography–mass spectrometry according to the protocol of Pan et al. (2010). SA (transition of 137/92.9) and JA (transition of 209.2/58.9) were detected in 50 mg of fresh -tobacco crude leaf extracts.

## Author Contributions

WD and QL designed the experiment. QL, YL, and YT carried out the experiment. QL wrote the manuscript. WD and JC improve the manuscript. All authors read and approved the final manuscript.

## Conflict of Interest Statement

The authors declare that the research was conducted in the absence of any commercial or financial relationships that could be construed as a potential conflict of interest.
