# Supplementary material for: Overexpression of NtWRKY50 Increases Resistance to Ralstonia solanacearum and Alters Salicylic Acid and Jasmonic Acid Production in Tobacco
Source: Front Plant Sci. 2017 Oct 11;8:1710. doi: 10.3389/fpls.2017.01710 (PMC5641554; doi:10.3389/fpls.2017.01710)
Supplement: Supplementary file 1 [file Data_Sheet_1.DOCX]

**

**

**Figure S1 The qRT-PCR analysis of deduced WRKY gene in response to various stresses.** Hydrogen peroxidase (H_2_O_2_), heat (38℃), cold (14℃), NaCl, Potato virus Y (PVY) or *Rhizoctonia solani* were applied on the leaves of 8-week-old WT plants. The total RNA was extracted from tobacco as described in material and methods. For white columns, *EF1α*, *ACT9* and *UBI3* were used as reference genes and for gray columns, the ubiquitin gene *UBI3* was used as reference gene. The transcript level in control was set to a value of ʻ1ʼ.

**

**

**Figure S2 Silencing of NtWRKY50 has no effect on tobacco susceptibility to *R. solanacearum* infection.** (A) Relative expression level of *NtWRKY50* in different NtWRKY50-silenced transgenic lines S1-S7. (B) Disease incidence of S2 and WT infected by *Ralstonia solanacearum*. Ten plants were used for each line and were root inoculated with 10 ml *R. solanacearum* inoculum. (C) Growth of *R. solanacearum* in roots of S2 and WT at 0, 1, 3 and 5 dpi. Plants were root wounded and inoculated as in B. Error bars indicate the SD of three independent experiments. No statistically significant (*t* test, *P* < 0.05) differences between S2 and WT were observed using SPASS version 13.0 software.

**Table S1** Primers used in this study

| Primers for QRT-PCR | | |
| --- | --- | --- |
| gene | Forward primer | Reverse primer |
| *UBI3* | GCCGACTACAACATCCAGAAGG | TGCAACACAGCGAGCTTAACC |
| *EF1α* | TGAGATGCACCACGAAGCTC | CCAACATTGTCACCAGGAAGTG |
| *ACT9* | AGGGTTTGCTGGAGATGATG | CGGGTTAAGAGGTGCTTCAG |
| *WRKY50* | GATCATATTCCAATCCCACT | TTGTCCCTTTCAACTCTTTT |
| *NtPR1A/C* | AACCTTTGACCTGGGACGAC | GCACATCCAACACGAACCGA |
| *NtPR1B* | AACCCATCCATACTATTCCTTG | GCCGCTAACCTATTGTCCC |
| *NtPR2* | TGATGCCCTTTTGGATTCTATG | AGTTCCTGCCCCGCTTT |
| *NtPR3* | CAGGAGGGTATTGCTTTGTTAGG | CGTGGGAAGATGGCTTGTTGTC |
| *NtACS1* | CATTAGCGAGGATTCGGAGTT | GTGGTGAATGAGGGATAGGA GA |
| *NtEFE26* | CGGACGCTGGTGGCATAAT | CAACAAGAGCTGGTGCTGGATA |
| *NtACC Oxidase* | GACAAAGGGACATTACAAGAAGT | GAGAAGGATTATGCCACCAG |
| *NtCAT1* | CAACTTCCTGCTAATGCTCCAA | TGCCTGTCTGGTGTGAATGA |
| *NtGST1* | AGCACCCTTACCTTTCCCTC | GCTTTCCTTCACAGCAGCATCA |
| *NtSOD* | TAGGATTTCATGGGTCATCT | TCCGTCTATTTGCTTGTTTA |
| Primers for construction of *NtWRKY50-*RNAi vector | | |
| fragment | Forward primer | Reverse primer |
| *NtWRKY50* RNAi forward target | cagtGGTCTCacaacATTCCAATCCCACTCCAA | cagtGGTCTCacaggATGAGAAGGGCTCTGGTG |
| *NtWRKY50* RNAi loop | cagtGGTCTCacctgcaggtctagtttttct | cagtGGTCTCagcccgggctctgtaactatc |
| NtWRKY50 RNAi reverse target | cagtGGTCTCagggcATGAGAAGGGCTCTGGTG | cagtGGTCTCatacaATTCCAATCCCACTCCAA |
|  |  |  |
| Primers for construction of *NtWRKY50-*overexpression vetor | | |
|  | Forward primer | Reverse primer |
| *NtWRKY50* | GGAAGATCTATGTCCTCTCCTTTACTTTCA | GGGTAACCTTATTATTAATGATGATGATGATGATGAAGGCGAAGGTTATGAAGG |
| Primers for *NtWRKY50-GFP* fusion vector | | |
|  | Forward primer | Reverse primer |
| NtWRKY50-GFP | TCCCCCGGGATGTCCTCTCCTTTACTTTCA | CGGGATCCCTAAAGGCGAAGGTTATGAAG |
